# Effects of Macro-/Micro-Channels on Vascularization and Immune Response of Tissue Engineering Scaffolds

**DOI:** 10.3390/cells10061514

**Published:** 2021-06-16

**Authors:** Nolan Wen, Enze Qian, Yunqing Kang

**Affiliations:** 1 Palm Beach County Campus, American Heritage Schools, Delray Beach, FL 33484, USA; nolanwen0@gmail.com; 2 Department of Ocean & Mechanical Engineering, Florida Atlantic University, Boca Raton, FL 33431, USA; eqian2016@fau.edu; 3 Department of Biomedical Science, Florida Atlantic University, Boca Raton, FL 33431, USA; 4 Department of Biological Science, Faculty of Integrative Biology PhD Program, Florida Atlantic University, Boca Raton, FL 33431, USA

**Keywords:** microchannel, porous scaffold, tissue engineering, immune response

## Abstract

Although the use of porous scaffolds in tissue engineering has been relatively successful, there are still many limitations that need to be addressed, such as low vascularization, low oxygen and nutrient levels, and immune-induced inflammation. As a result, the current porous scaffolds are insufficient when treating large defects. This paper analyzed scientific research pertaining to the effects of macro-/micro-channels on the cell recruitment, vascularization, and immune response of tissue engineering scaffolds. Most of the studies contained either cell culturing experimentation or experimentation on small animals such as rats and mice. The sacrificial template method, template casting method, and 3D printing method were the most common methods in the fabrication of channeled scaffolds. Some studies combine the sacrificial and 3D printing methods to design and create their scaffold with channels. The overall results from these studies showed that the incorporation of channels within scaffolds greatly increased vascularization, reduced immune response, and was much more beneficial for cell and growth factor recruitment compared with control groups that contained no channels. More research on the effect of micro-/macro-channels on vascularization or immune response in animal models is necessary in the future in order to achieve clinical translation.

## 1. Introduction

Despite the plethora of studies on porous scaffolds in many tissue engineering fields and the success achieved, there are still major challenges to be addressed, such as low vascularization, low oxygen and nutrient levels, and immune-induced inflammation; the size of the regenerated tissue has only reached a few hundred micrometers. Recent studies have shown that the incorporation of macro-/micro-channels into a porous scaffold may increase vascularization and allow oxygen and other nutrients to be distributed equally throughout the scaffold in regenerating larger tissue constructs [1]. The purpose of this literature review is to critically analyze scientific research pertaining to the effects of macro-/micro-channels on cell recruitment, vascularization, and immune response of tissue engineering scaffolds.

### 1.1. Tissue Engineering

With the increased demand for regenerative medicine, tissue engineering products are widely used in repairing defective tissues that arise through accidents and injuries and treating diseases that cannot be otherwise cured through ordinary medicines [2]. The entire industry is moving towards the goal of creating functional substitutes for damaged tissues [3]. Tissue engineering scaffolds, which are made of polymers, ceramics, and composite biomaterials, are of great interest as implanted scaffolds due to their wide range of properties and tunability to match the needs of forming diverse structure [4,5]. However, insufficient vascularization and inappropriate immune response weaken the regenerative function of implanted scaffolds [6,7,8,9].

### 1.2. Vascularization in Tissue Engineering

Currently, many types of engineered tissues, such as skin, bone, ligament, adipose, and muscle have been studied in vitro; however, the size of the tissue has only reached a few hundred micrometers [10]. The limitation of in vitro engineered tissues is based on the inability to create blood vessel systems [11,12]. In vivo, blood vessel system supplies the nutrients and oxygen to nearly all tissues, and the maximum distance between the capillaries is 200 μm [13,14]. Vascularization became a major obstacle in maintaining the regeneration of tissues within tissue engineering [15].

The developed strategies for enhancing rapid vascularization included: delivering angiogenic growth factors [6,16] and co-cultured exogenous endothelial cells and pericytes [17,18]. Although on the surface of the scaffold those methods can help to initialize angiogenesis, depending on the structure of the porous scaffolds, the situation in the deeper area or the central zone still cannot be improved [6]. Although interconnected pores are required for the migration of cells and the transport of nutrients and growth factors [19,20], the pores structure is not sufficient for the vascular system to provide the in-growth of host vasculature and nutrient diffusion [21,22]. The shortage of oxygen and nutrients will greatly delay the tissues regeneration. In this case, the researchers focused on the creation of bionic blood vessel channels in the scaffold.

### 1.3. Immune Response in Tissue Engineering

Immune response is a double-edged sword. While providing high-efficiency protection for humans, it also brings huge clinical challenges to the prognosis of surgery or scaffold implantation [23]. Following the scaffolds implantation, macrophages in the human immune system will start working immediately. Macrophages, as the early-appeared immune cells in the injured site, are involved in both advancing inflammation and promoting tissue repair and regeneration [24,25,26]. Generally, there are two types of macrophages: classically-activated phenotype (M1), which may cause inflammation and swelling and impede regeneration within the scaffold [27], and the alternatively-activated phenotype (M2), which may promote tissue regeneration [28,29,30]. The cumulative studies verified that topological attributes of scaffolds including fibers, pores, and channels can differentially promote the macrophage polarization [31,32]. Changing the pore size of a bi-phase calcium phosphate ceramic scaffold induce the macrophages to release different cytokines [29]. Modifying the titanium surface promoted the polarization of macrophage [33]. Currently, researchers in various fields of tissue engineering are using channeled scaffolds to regulate immune responses, especially macrophage polarization. In this paper, we review the importance of channels in a porous scaffold for vascularization and immune response.

## 2. Macro-/Micro-Channels in Porous Scaffolds

### 2.1. Literature Search

In this minireview, we use PubMed to conduct a systematic literature search. Keywords used for the search are “Channel, Vascularization, Tissue Engineering, Scaffold”, “Channel, Vascularization, Tissue Regeneration, Scaffold”, and “Channel, Immune Response, Tissue Engineering, Scaffold”. Only original research studies published within the last 10 years evaluating the effect of channels on cell response, vascularization, and/or immune response were selected. Review articles, materials research without biological evaluations, and research focused on simulation or in vitro research models that do not target tissue regeneration applications were excluded. Table 1 summarizes the basic information of some related studies including the following: scaffold material, channel type, fabrication method, target application, and research model for each study. A narrative review was provided for each article in terms of the detailed methodology and the effect of channels on cell recruitment, vascularization and immune response as well as the significance of the research findings. The content was organized according to the target applications.

### 2.2. Methods of Channel Fabrication

The three main methods used to create macro-/micro-channels within porous scaffolds are: (1) mechanically removable template method, (2) sacrificial template method, and (3) 3D bioprinting. The mechanically removable template method includes the use of templates or removable rods to create macro-channels. This method has shown the potential to create channels in the scaffolds and provide structure for cells within the channels [47,48]. Secondly, the use of a removable template spacer is instrumental in the sacrificial template method. Usually, two materials are used; the material of the sacrificial template would have a lower melting or dissolving point than the material of the scaffold. After the creation of the template, the template will either be heated up or dissolved, leaving branched macro-/micro-channels within the scaffold [45,49]. The use of 3D bioprinting is an extremely promising method for creating macro-/micro-channels within scaffolds. Unlike the previous two methods which require many steps to create macro-/micro-channeled scaffolds, the newly developed 3D bioprinting techniques can significantly simplify the design and development process [50,51]. In addition to the method of creating channels, the number of channels within a scaffold and the sizes of the channels may influence the effectiveness of the scaffold. If the incorporation of macro-/micro-channels within a scaffold increases vascularization within the scaffolds and allows oxygen and other nutrients to be distributed equally throughout the scaffold, this will bring promise in regenerating even larger tissues [48].

### 2.3. Channel for Vascularization and Immune Regulation

#### 2.3.1. Prevascularization and Blood Perfusion

**Figure 1:**
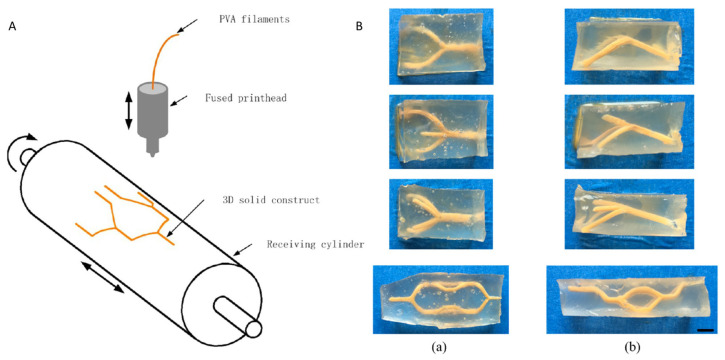
Schematic for the printing of the three-dimensional solid sacrificial template (**A**). (**B**) Perfusion of the fabricated connected networks. (**a**) Front view; (**b**) side view. Scale bar is 3 mm. Reprinted (adapted) with permission from [42]. Copyright © 2016, American Chemical Society.

Prevascularization is one of the most significant factors that greatly influence the scaffold’s functions. Li et al. combined the sacrificial template and bioprinting method to create channels in the scaffold [42]. They created a sacrificial molding technique to fabricate scaffolds with 3D channels (Figure 1). Poly(vinyl alcohol) (PVA) filament was 3D-printed and used as the sacrificial material. The PVA sacrificial material was printed onto a cylinder and after printing, it was taken off the cylinder and put into a container containing a mixture of microbial transglutaminase (mTG) and gelatin. After the mTG and gelatin cross-linked, the inner PVA template was dissolved by phosphate-buffered saline (PBS) to form a 2.2–3 mm wide channel inside of the hydrogel. The authors seeded human umbilical vein endothelial cells (HUVECs) into the channels and cultured the cells to let them attach to the channels. The results showed that after 2 days of culturing, cells were beginning to fill up areas previously untouched, especially in the middle of the channel where there previously were no cells. Two more days of culturing later, the HUVECs were distributed uniformly through the entire channel, showing that the materials and processes used to create the channels were nontoxic and promoted endothelial cell attach for prevascularization.

Similarly, Mirabella et al. used 3D printing to fabricate sacrificial glass template to make patterned channels within a fibrin patch [52]. The channels with endothelial cells facilitated rapid invasion and integration of small collaterals from the host to generate perfused, functional blood vessels. The long, centimeter-scale channels orientated in parallel directly revascularized ischaemic tissues through the endothelialized channels. Kinstlinger et al. also used the concept of sacrificial templates to fabricate perfusable vascular networks in engineered tissues [53]. They showed that the algorithmically generated dendritic vessel networks and other complex hierarchical networks can help sustain thick and densely cellularized engineered tissues and assist in the interrogations of the interplay between mass transport and tissue function. Bagrat Grigoryan et al. used the projection stereolithography method to create distinct vascular networks that were embedded in a photopolymerizable hydrogel [54]. Their work showed that the entangled functional vascular networks from space-filling mathematical topologies can support the studies of fluid mixers, valves, intervascular transport, nutrient delivery, and host engraftment. They deployed the structured biodegradable hydrogel carriers in a rodent model of chronic liver injury. The results showed the potential translational utility of the multivascular networks in tissue regeneration.

#### 2.3.2. Neo-Vascularization in Large Tissue Construct Engineering

**Figure 2:**
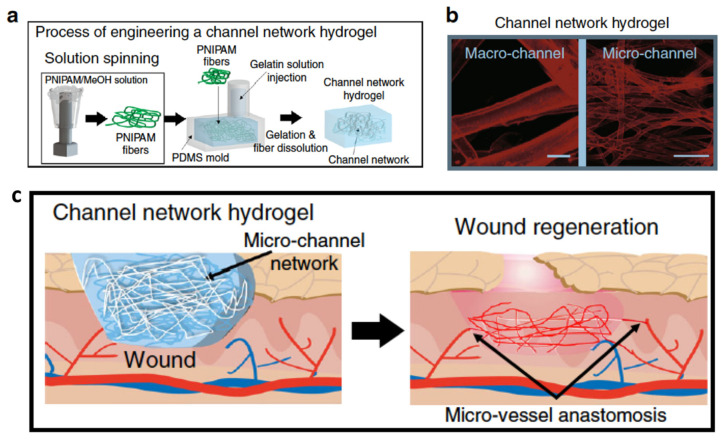
(**a**). Schematic illustration of the procedure to produce poly(Nisopropylacrylamide) (PNIPAM) fibers, then channel networks, in a hydrogel within a PDMS mold. (**b**). Confocal visualization of micro- or microchannel networks in hydrogels with their channel diameter distribution. Channels were perfused with FluoSpheres (45 nm, red). Scale bar = 100 μm. (**c**). A Schematic illustration of hydrogel implantation into a wound site post-full-thickness defect of mouse dorsal skin with the discovered regeneration process (top box). Photographs of wound healing sites at day 14 post-implantation (bottom row). Reprinted (adapted) with permission from [43] under a Creative Commons Attribution 4.0 International License.

There is an urgent need for scaffolds that support large, critically sized tissue formation in the field of regenerative medicine. The major obstacles in reaching this goal are the delivery of oxygen and nutrients throughout the engineered tissue and host tissue integration and vascularization post-implantation. To address this long-standing issue, angiogenesis induction into damaged sites is highly required through the potential of channels, especially in a large tissue construct. Lee et al. fabricated 3D channel network hydrogels through the following process: (i) poly (N-isopropylacrylamide) (PNIPAM) was solution-spun to produce fibers [43]. (ii) PNIPAM fibers were embedded in a polydimethylsiloxane (PDMS) mold, then an enzyme-crosslinkable gelatin solution was poured onto them, followed by gelation cross-linking. (iii) The temperature-dependent water-solubility of PNIPAM enabled the fibers to dissolve by washing with water perfusion when the temperature was lowered below 32 °C, representing a non-toxic, organic solvent-free process (Figure 2). As a result, void channel networks were produced after fiber dissolving and perfusion washing. The average diameters of macro- and micro-channels were 150.46 ± 57.02 μm and 16.37 ± 7.76 μm (mean ± SEM), respectively. At day 14 post-implantation in the mouse model of severe hindlimb ischemia, the microchannel group showed clear microvessel invasion from the host femoral artery into the hydrogel in contrast to the other test groups, i.e., the control (saline and without channel) and macrochannel groups. The indicators of the functional vascular structure, i.e., the total branching length, number of branches and junctions, and fractional area in the microchannel hydrogel group were found to be significantly higher than those in the other test groups, at a level similar to that of the normal limb.

Rnjak-Kovacina et al. created hollow channels in a silk scaffold to address the issue of insufficient vascularization [10]. Within this paper, in vitro and in vivo experiments were conducted. To understand the effects of channels with a diameter of 508 µm on cell infiltration and oxygen/nutrient delivery, cells were seeded into a scaffold with a diameter of 12 mm and a height of 4 mm. For the in vivo experiments, channeled (254 µm and 508 µm diameter) and unchanneled scaffolds were used to observe vascularization and immune response. Two weeks after implantation into mice, both the 254 µm and 508 µm channeled silk scaffolds had a higher rate of vascularization compared to the non-channeled group. After two weeks the blood vessels were already functioning and mature. The implantation of the silk scaffolds elicited a standard immune response as there was very little inflammatory response. After two weeks of implantation, all groups contained immune cells, but after 4 weeks, the majority of cells in the scaffold were dermal fibroblasts (creating connective tissue).

Neo-vascularization is very important for tissue regeneration within porous scaffolds. Varoni et al. [44] created three types of microchannels by the electrochemical replica-deposition of chitosan. All three of the groups contained microchannels with a diameter of 500 µm, but the spacing between the microchannels differed. The distances were 600, 700, and 900 µm. The group with the distance between the microchannel as 700 µm had the most favorable mechanical properties so it was the group used in the in vivo experimentation. The scaffolds were implanted into six-week-old mice. After 3 weeks of implantation into rats, the capillary density of the micro-patterned group was higher than that of the random-pore group. After 6 weeks, the difference in capillary density was statistically significant. The effect of degradation did not lead to any humoral immune response, whether at 3 weeks or at 6 weeks after surgery. There were no detectable signs of inflammation or foreign body response. Main perspectives include the application of these tailored scaffolds in regenerative medicine, where neovascularization is mandatory, with particular attention for those medical fields in which tissue needs to be regenerated in a specifically oriented manner. Their practical applications will include, indeed, joint regeneration in orthopedic medicine and periodontal regeneration in periodontology, in order to promote both neovascularization and the growth of spatially oriented ligament fibers.

#### 2.3.3. Immune Regulation

To observe whether channels can regulate immune response and obtain functional vascular structures for ischemia treatment, Lee et al. created perfusable microchannel networks in a hydrogel [43]. In mouse and porcine models of hindlimb ischemia, its functional vascular structure with microchannel size promoted proangiogenic M2 polarization of macrophages and consequent functional endothelial cell (EC) recovery. It was observed that the regenerative M2 polarization of macrophages was more dominant in the microchannel group than in the macrochannel group, contrary to the destructive M1 polarization pattern, indicating an M2 polarization mediator in the microchannel hydrogel rescue mechanism. The gene expression of a skeletal muscle marker was higher in the macrochannel group than in the microchannel group, indicating an invasion of skeletal muscle with increased channel size. The test groups were implanted in a well-established mouse model of wound healing to examine if the microchannel network hydrogel can be applied in another model of ischemic damage with the presence of neighboring host vasculature. The implantation of the microchannel group was found to accelerate the wound closure process as compared to the macrochannel group and the w/o channel hydrogel group. CD 31 expression (EC marker) and IL10 secretion (M2 polarization) were higher in the microchannel group compared to the macrochannel group but the differences of other factor amounts between the micro- and macro-channel groups were insignificant. This means that it is difficult to discern the effect of channel size on the wound healing model as compared to the severe hindlimb ischemia model. Given that both hindlimb ischemia and wound closure models are exposed to insufficient oxygen supply with a rich presence of neighboring host vessels, three other in vivo models (implantation to the omentum, normal limb, and subcutaneous tissue) were employed to determine the mechanistic roles of the ischemic condition and neighboring blood vessels. Although these three models were not subjected to ischemia, the number of neighboring vessels is in decreasing order from rich (omentum), to middle (normal limb), and finally, to poor (subcutaneous) levels: (i) the microchannel group showed the most effective perfusion from host vessels to the implanted sample and the richest population of CD31+ cells; (ii) however, these levels in the microchannel group decreased markedly from the omentum model to the normal limb, and lastly, to the subcutaneous model; and (iii) even though the perfusion performance was most efficient in the microchannel group of the omentum model among all non-ischemic test groups, its level appeared to be lower than that of the microchannel group in the hindlimb ischemia model. The study showed the significant function of an implantable hydrogel with a perfusable microchannel network that is effective for the treatment of ischemic/inflammatory disease by promoting angiogenesis and M2 polarization of the infiltrated monocytes/macrophages. The microchannel structure without loading any cells or therapeutic molecules enabled vascular perfusion throughout the hydrogel implant and promoted macrophage polarization.

### 2.4. Channel for Tissue Engineering through Vascularization and Immunomodulation

#### 2.4.1. Bone Tissue Engineering

3D bioprinting has been showing great promise in the advancement of developing functional tissue/organ replacements [51,55,56,57]. However, to realize the true potential of 3D bioprinted tissues for clinical use, the fabrication of an interconnected and effective vascular network is required. Holmes et al. [34] was successful in designing and printing a series of novel 3D bone scaffolds that had structures supporting bone formation and highly interconnected 3D microvascular mimicking channels, for efficient and enhanced osteogenesis as well as vascular cell growth. The 3D scaffold designs were 3D printed layer by layer with polylactic acid (PLA) [34]. The diameter of the horizontal and vertical channels was between 250 and 500 μm. In addition, nano-hydroxyapatite (nHA) was successfully conjugated onto scaffolds post fabrication to further improve osteogenic properties. Enhanced human mesenchymal stem cell (hMSC) adhesion and growth were observed on scaffolds with small microchannels (250 μm) and nHA modification while scaffolds with large channels (500 μm) promoted the greatest human umbilical vein endothelial cell (HUVEC) growth. The scaffolds designed and fabricated in this study had physical properties comparable to bone fracture regimes, and flow measurements revealed that the designed microchannels had similar flow characteristics to native blood vessels. They may provide a powerful construct for further in vitro experiments where multiple cell types are co-cultured, or for future in vivo study.

**Figure 3:**
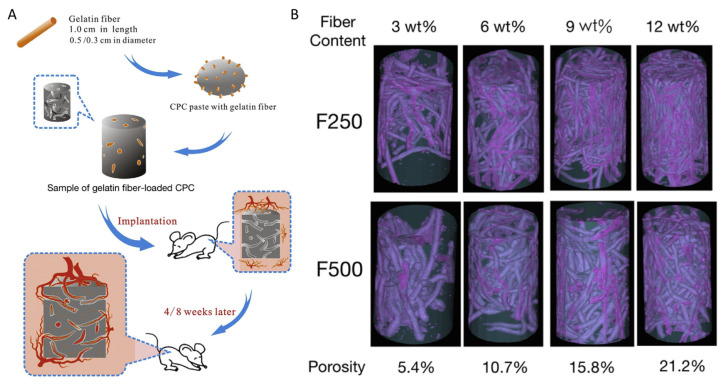
Schematic for the experimental protocol of the vascularization strategy with an in situ formed channel in CPC structure. Schematic of the vascularization strategy within channels of CPC scaffold: (**A**). Different channel diameters induced the different expression behaviors for growth factors; then induced the different vessel forming. (**B**). Micro-CT analysis of CPC-fiber scaffold: 3D reconstruction images (F250/F500: different diameter of fiber; X wt.%: different content of fiber in CPC). Reprinted (adapted) with permission from [35]. © 2016 Elsevier B.V. All rights reserved.

Although calcium phosphate cement (CPC) is a promising material for bone repair therapy, its slow biodegradation and insufficient vascularization in constructs negatively impact its clinical application [35]. In experiments conducted by Yu et al. [35], interconnected hollow channels in CPC were formed after the dissolution of micro (255 ± 8 µm) and macro (507 ± 9 µm) gelatin fibers in vivo. There were 5 groups in this study: the F250 and F500 groups with fiber groups in which the fibers were not removed, the F250 and F500 groups with channels in which dissolution of the fiber occurred, and the control group in which no fiber was added at all. Experiments were conducted on rats and results were analyzed four and eight weeks after implantation. When the calcium phosphate cement was implanted in vivo, the vascular network in the cement was anastomosed with blood vessels and accelerated vascular infiltration throughout the entire construct. The results showed that tissue growth did not occur within the control group but did occur rapidly in the groups containing channels. Although both channel groups accelerated vascularization, different channel sizes did induce different vascularization behaviors in vivo (Figure 3). In the F250 group with channel, the angiogenic factors HIF1α, PLGF, and migration factor CXCR4 released benefited the formation of small vessels in diameter of 5–10 µm at 4 weeks and 5–18 µm at 8 weeks. On the other hand, in the F500 group with channels, different angiogenic factors (VEGF-A) were released, promoting the formation of large-sized vessels in diameter of 4–20 µm at 4 weeks and 5–45 µm at 8 weeks. The difference in vessel diameter between the two groups was statistically significant, proving the size of the channel has an effect on vascularization. Inflammation did occur in the experimental groups after the cement was implanted into the rat, however, it did not have a significant effect on the effectiveness of vascularization within the rats. After eight weeks, the amount of granulation tissue increased in both the F250 and the F500 groups and inflammatory cells were not observed.

Modulation of the physicochemical properties of scaffolds can help in recapitulating the in vivo microenvironments of damaged tissues and boost the endogenous regenerative capacity. Won et al. prepared PCL scaffolds with microchanneled structures via 3D printing [36]. The authors used camphene in the PCL to form the microchannels because it mixes easily with other materials, has a high volatility, and a low temperature of sublimation. The camphene/PCL scaffold was printed into a 37 °C ethanol/DW (distilled water) solution, allowing the pore structures to be uniform. The average pore size in the scaffold was 12.9 µm. There were two experimental groups within this experiment, the ‘Non’ group, which contains no channels and the µCh group which contained microchannels. Experiments were done in vivo in rats. Compared to the ‘Non’ group, the ‘µCh’ group had a much higher rate of vascularization. After 6 weeks of implantation in rats, they observed X-ray and micro-CT images and observed hard tissue formation within rats containing the ‘µCh’ scaffold in the defect area. In the contrary, hard tissue formation was limited in the ‘Non’ group containing no microchannels. In addition, new bone volume, surface area, density, and new bone formation in the defect area after 6 weeks were all substantially higher in the ‘µCh’ group than in the ‘Non’ group. Based on the results, the addition of channels to scaffolds greatly increased vascularization in the defect area. Won et al. realized during experimentation that VEGF was released at significantly higher levels in the ‘µCh’ group than the ‘Non’ group [36]. According to Yu et al. [35], the angiogenic factor VEGF promoted the growth of larger blood vessels. As a result, it would be reasonable to predict that the blood vessels in ‘µCh’ were larger and more stable than the blood vessels in the ‘Non’ group.

Besides angiogenesis, Won et al. also investigated the effect of µCh on immune response [36]. Twenty-four hours or so after an injury or implantation, neutrophils migrate to the wound. These neutrophils have the ability to form neutrophil extracellular traps (NET) which can stimulate the further immune response. In the ‘Non’ group, NETs were present while they were not present in the ‘µCh’ group. This shows the implementation of channels decreases immune response and allows for tissue repair because NETs may stimulate more immune response, which would slow down the tissue repair process. In addition, the number of NOS2 positive cells (M1 macrophages) was two times greater in the ‘Non’ group (15%) than the ‘μCh’ group (8%). However, the number of arginase positive cells (M2 macrophages) was much higher in the ‘μCh’ group compared to the ‘Non’ group. M1 macrophages promote inflammation with pathogen-killing abilities while M2 macrophages promote cell proliferation and the repair of tissue. This proves that channeled scaffolds attract macrophages and growth factors beneficial to tissue growth while scaffolds without channels limit tissue regeneration.

During normal skeletogenesis, canalsin developing hypertrophic cartilage play a major role in the process of endochondral ossification [37]. Inspired by this developmental feature, the objective of the study conducted by Sheehy et al. was to promote endochondral ossification of an engineered cartilaginous construct via modifying the scaffold architecture [37]. Their hypothesis was that the introduction of channels into mesenchymal stem cells (MSC)-seeded hydrogels would firstly facilitate the in vitro development of scaled-up hypertrophic cartilaginous tissues, and secondly would accelerate vascularization and mineralization of the graft in vivo. MSCs were put into hydrogels either containing or not containing microchannels. Agarose cell suspension was put into a stainless-steel mold to make the non-channeled constructs. To make the channeled constructs, a pillared polydimethylsiloxane arrayed structure was inserted into a cylindrical construct. The channels had a diameter of 500 µm and a spacing of 1 mm. The channeled and solid constructs were cultured for 5 weeks in chondrogenic conditions, followed by 1 week of culturing in hypertrophic conditions, and then subcutaneous implantation into nude mice to be retrieved at 4 weeks and 8 weeks. Upon retrieval from the back of nude mice, macroscopically the channels appeared to the filled with a reddish, well vascularized tissue [37]. This vascularized tissue spread through the depth of the channels. The solid constructs did not appear to be vascularized. Hematoxylin and eosin (H&E) staining of the solid construct group did not show bone formation 4 weeks post-implantation. H&E staining of channeled constructs at 4 weeks post-implantation demonstrated woven bone formation within the channels, surrounding a marrow component consisting of a mixture of hematopoietic foci and marrow adipose tissue. At 8 weeks post-implantation, no bone formation was evident in solid constructs, whereas within the channels of channeled constructs lamellar-like bone was evident, with the appearance of osteocyte-like cells embedded within the bone matrix surrounding a marrow component. The study reinforces the importance of optimizing the architecture of engineered constructs targeting bone tissue regeneration, even if this is achieved via an endochondral pathway as opposed to the traditional intramembranous route.

**Figure 4:**
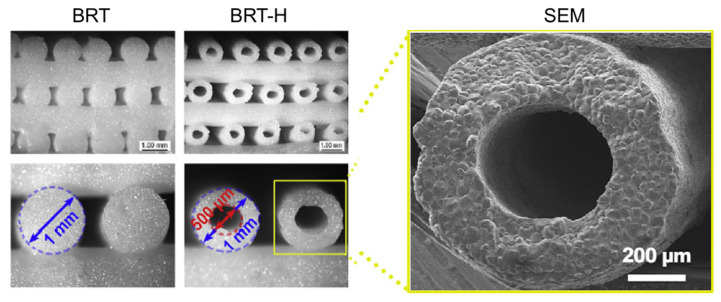
The structure of the BRT-H scaffolds with about 500 μm inner channels. Reprinted (adapted) with permission from [38]. © 2017 Published by Elsevier Ltd.

Segmental bone regeneration is very difficult because of the low degree of vascularization. To solve this problem, Zhang et al. fabricated hollow-pipe-packed silicate bioceramic (BRT-H) scaffolds using a coaxial three-dimensional (3D) printing technique [38]. By utilizing a modified core/shell printer nozzle and a modulated viscoelastic bioceramic paste, the authors were able to print hollow struts with an external diameter of 1 mm and internal diameter of 500 µm (Figure 4), resulting in a BRT-H scaffold with a compressive strength as high as 26 MPa. BRT scaffolds without hollow pipes and beta-tricalcium phosphate (β-TCP) scaffolds as controls were fabricated using a conventional printer nozzle. To investigate the effect of the BRT-H scaffolds on the vascularization and regeneration of large bone defects, the rabbit radius segmental defect model was employed to evaluate both the angiogenesis and osteogenesis processes. After implantation in the segmental bone defects for 4 weeks, the angiogenic ability was first detected, and the microfiber perfusion blood vessels in these scaffolds were reconstructed via micro-CT analysis. Numerous blood vessels were observed on the inside of the hollow pipes in the BRT-H group. In addition, the amount of blood vessels in the BRT-H scaffolds was significantly higher than that in the BRT group without the hollow pipe structure. The results showed that the hollow pipes in the scaffolds and BRT bioceramic synergistically enhanced the angiogenic process during the early stages of bone regeneration. Moreover, new bone tissues were found to grow within the hollow pipes at week 4 in the BRT-H group.

**Figure 5:**
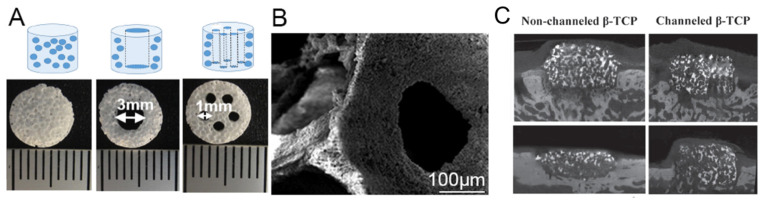
Schematic illustration and digital morphologies of three types of β-TCP scaffolds’ structure, (**A**) without channel, with singular 3 mm diameter channel and with five 1 mm diameter channels, (**B**) SEM image showed interconnected pores and local strut surface. Reprinted (adapted) with permission from [48]. Copyright © 2016, American Chemical Society. (**C**) Micro-CT images show that channeled scaffolds can maintain the height of regenerated bone, but scaffolds without channels degraded and lowered the height. Reprinted (adapted) with permission from [39] © 2016 WILEY-VCH Verlag GmbH & Co. KGaA, Weinheim.

A major challenge for achieving successful vertical alveolar bone augmentation using synthetic scaffolds is insufficient vascularization. Due to the slow ingrowth of host vasculature coronally from the bone bed, typically, there is limited bone formation in the coronal portion of the implanted grafts, which could compromise the success rate of subsequently placed titanium dental implants. Wang et al. fabricated macroporous beta-tricalcium phosphate (β-TCP) scaffolds with multiple vertical hollow channels to act like blood vessels for nutrient diffusion and cell migration [39,48]. The scaffold was created with β-TCP and contained five vertical macro-channels (Figure 5A,B). A template casting method was used to create the β-TCP scaffold. The authors also incorporated a bone-forming peptide (BFP-1) to accelerate bone and blood vessel formation in some of the groups. Beagle dogs were used as bone augmentation models. Three experimental groups were designed as follows: (a) channeled scaffolds with BFP-1; (b) non-channeled scaffolds with BFP-1; (c) no implant control. The scaffolds were implanted in bone defects (8 mm in diameter and 2 mm in depth) in the mandibular bone of beagle dogs and observed for 4 and 12 weeks. The authors observed that vascularization had occurred much more rapidly in the group containing channels than the groups without channels and the control group without any scaffold. At 4 weeks, there was already a significantly higher amount of blood vessel formation in the scaffolds containing microchannels. After 12 weeks of implantation, the height of the channeled scaffold group (4.75 ± 0.57 mm) was significantly greater than the group without channels (2.16 ± 0.51 mm). This further proves that channels improve vascularization, allowing both the scaffold and bone to maintain its shape (Figure 5C). These results in animals showed that channeled scaffolds could hold the height of new regenerated bone and promote vascular growth on bone augmentation. However, the channeled scaffolds had no significant influence on bone density compared with the non-channeled scaffolds. The new regenerated bone was mainly of immature bone.

#### 2.4.2. Myocardial Tissue Engineering

The pre-vascularization of myocardial tissue represents a major challenge in cardiac tissue engineering. Fang et al. employed the sacrificial template method to create channels within the biomimetic scaffold [40]. The sacrificial template used to make the channels was a carbohydrate. A mixture consisting of one gram of glucose, two grams of sucrose, and twelve grams of maltose was heated, and 3D printed at a temperature of 120 °C. After the sacrificial template was created, it was submersed into a poly(lactic-co-glycolic acid) (PLGA)/chloroform mixture. NaCl particles were also mixed into the PLGA solution to create microholes on the PLGA membrane. Finally, the sacrificial template was immersed in deionized water, dissolving the template. The resulting scaffold contained channels between 580 and 744 μm with microholes incorporated on the wall of the channels. Cardiomyocytes and endothelial cells (HUVECs) were independently seeded into the porous zone and channel networks of the scaffold through perfusion, providing heterogeneous distribution of cardiomyocytes and endothelial cells. After 2 h, the endothelial cells were found around the bifurcating channels and adhered to the channel walls. After 7 d of in vitro culture with perfusion, a confluent endothelial cell lining was observed on the luminal network surface in the hollow channel. Interestingly, sometimes, endothelial cells appeared to migrate through the 10–50 μm micro-holes of the branch channel into the scaffolds and formed single or multicellular sprouts from the inner luminal endothelium. The authors were able to achieve heterogeneous cell distribution with myoblast cells distributed throughout the scaffold and around the channel network, whereas endothelial cells adhered to the inner lumen of the network. Two distinguishable features of these scaffolds were its orientated micropores and its perfusable channels. Both of these features played a fundamental role in cell orientation, tissue maturation, and vascularization of the engineered tissue.

Pre-vascularization plays a key role in reconstructing dense, metabolically active myocardial tissue and its integration with the host myocardium post-implantation. Zieber et al. used a laser piercing technique to create channels inside 2 mm thick alginate scaffolds [41]. The channels created were parallel to each other and had a 200 μm diameter and a channel-to-channel distance of 400 μm. Cells were seeded sequentially onto the scaffolds in the following order: firstly, HUVECs were seeded and cultured for three days, then neonatal rat cardiomyocytes (CMs) and cardiofibroblasts were added with a final cell ratio of 50:35:15, respectively, and the scaffolds were cultured for seven more days. A vessel-like network was observed within the cell constructs, wherein HUVECs were found to organize around the channels in a multilayer manner, while the CMs were located in-between the channels and exhibited the characteristic morphological features of a mature cardiac fiber. The channeled and non-channeled acellular scaffolds with the affinity-bound basic fibroblast growth factor were implanted in mice subcutaneously. After 4, 6, and 8 weeks, the scaffolds were removed from the mice and analyzed. After implantation, the authors observed that cells entered the channeled scaffolds much more easily than the non-channeled scaffold. Cells were found in the interior section of the channeled scaffold while they were only on the periphery of the non-channeled scaffold. This shows how the incorporation of channels in scaffold allows for the easy movement of nutrients into the cell, providing a healthy environment for cell growth. In addition, there was also a statistically greater amount of blood vessels in the channeled implants than the non-channeled implants at weeks six and eight, proving that channels improved vascularization. In the present study, both the in vitro and in vivo results point to the importance of micro-channel fabrication as a promoter of scaffold vascularization.

#### 2.4.3. Nerve Tissue Engineering

For nerve injury, the standard clinical intervention, end-to-end anastomosis is only suitable for defects shorter than a few millimeters [58]. For complicated nerve injury autologous transplantation is considered as the ‘gold standard’ in the current nerve repair practice [59,60]. However, it also has limitations such as a secondary operation, limited availability and the risk of neuroma formation [61,62]. Researchers believe that nerve conduits have emerged as promising substitutes for autografts [46,63]. Zhu et al. created an extracellular matrix scaffold with instructive niches for oriented nerve regeneration [45]. The decellularized ECM channeled scaffold (ECM-C) was prepared by using PCL microfibers and rat implantation. The final channel diameter was 146.6 ± 6.9 μm with safety DNA concentration as 32.3 ± 9.1 ng/mg [64]. The experiment of rat artery implantation proved that ECM-C has excellent vascular regeneration ability. The researchers designed a 3-month rat sciatic nerve regeneration experiment and modified the ECM-C as tubular scaffold. The nerve ECM-C scaffold and control scaffolds were implanted into 15 mm rat sciatic nerve defects. After 3 months, the ECM-C scaffold showed that it can promote the nerve remodel, and the neo-nerves had the similar morphology and color with the native nerves. H&E staining showed that a high density of myelinated fibers existed in the both lumen and microchannels of ECM-C, but in the control group, only sparse immature nerve fibers distributed within the lumen of the scaffold. At the same time, the degree of vascularization in the ECM-C group was significantly higher than that in the control group, especially in the wall microchannels. The authors believe that the ECM-C scaffold showed the potential for directed nerve regeneration to repair nerve defects.

**Figure 6:**
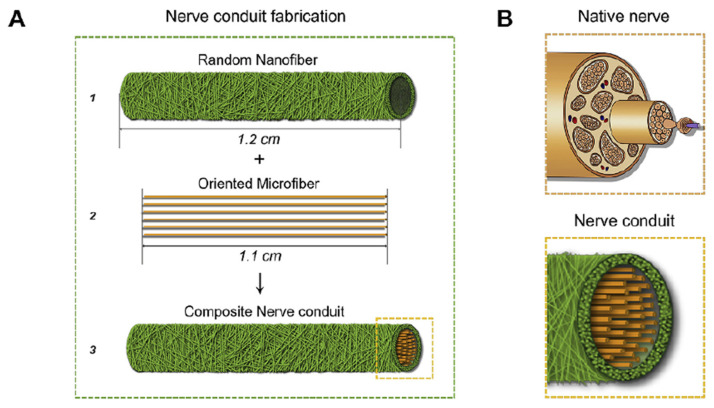
Preparation of nerve conduit and characterization of macrophage polarization on random nanofiber and oriented microfiber substrates. (**A**,**B**) Schematic illustration of natural nerve structure and biomimetic nerve conduit structure along with key fabrication steps. The conduits of A1 and A3 were respectively used for control and experimental groups. Reprinted (adapted) with permission from [46] © 2021 Elsevier Ltd. All rights reserved.

Following nerve injury, macrophages are the early-appearing immune cells in the injured site [65,66]. Dong et al. created a scaffold which is composed of oriented microfiber cores and random nanofiber sheaths to indirectly promote nerve regeneration by promoting the polarization of macrophage to M2 phenotype [46]. As shown in Figure 6, the exterior shell was made by random PLCL nanofibers with 0.7 ± 0.2 µm in diameter, the longitudinally oriented PDS microfibers with 27.1 ± 3.9 µm in diameter were filled in the lumen, and the final diameter of the tube was 240 ± 12 µm with inter-fiber pore size of 4.1 ± 0.9 µm. In the in vitro experiment, murine macrophages (RAW 264.7) were cultured on the oriented PDS microfiber and randomly organized PLCL nanofiber. The result showed that oriented PDS microfiber can promote the polarization of the macrophage to an M2 phase while the macrophages expressed a high level of M1 in the control group and the organized PLCL nanofiber group. Under the influence of different phenotype of macrophage, the Schwann cells (SCs) migration length, branching length, and the amount of branched SCs were all enhanced by oriented PDS. In subsequent in vivo experiments, the rat sciatic nerve defects model proved that the oriented microfiber therapeutic effect was close to autologous transplantation and significantly better than the random nanofiber group. Zhu et al. [45] point out that the oriented microfiber can facilitate the recruitment of macrophages and promote the macrophage to polarize to M2 phenotype, which subsequently enables the migration, proliferation of SCs extension for better restoration of nerve function and regeneration of nerve tissue.

## 3. Discussion

### 3.1. Summary of Topic

Over the past 50 years, tissue engineering has evolved into an extremely promising method for treating wounds needing tissue repair or regrowth. Although the use of scaffolds in tissue engineering had shown positive results in cell culturing and small organism experimentation, there are still concerns that they may not be sufficient in larger defects or organisms. This is because scaffolds in the larger defects are unable to circulate blood and necessary nutrients throughout the defect, especially in the central areas. Recently, new research has been done on the incorporation of macro-/micro-channels into these scaffold structures to promote vascularization throughout the construct, promote cell recruitment, and initiate the positive immune response. After analyzing and comparing all the studies pertaining to this topic, the incorporation of channels seems like an effective solution to the current problems. A common theme that was portrayed throughout all research was that the development of macro-/micro-channels within scaffolds had a dramatic increase in vascularization compared to the control group with no channels or even other groups with pores instead of channels. Within these studies, many of the papers did not address the effect of channels on immune response and cell/growth factor recruitment; however, the available studies did show that channeled scaffolds generate more positive immune response and improved cell/growth factor recruitment.

### 3.2. Application

Most of the articles addressed in this literature review attempt to regenerate bone, myocardial tissue, and nerve tissue as these tissues are highly vascularized. Although the authors of these studies mostly experimented on these types of tissue, tissue regeneration can be applied to many different types of tissue, such as liver, skin, and even brain tissue through functional channels. Today, the use of tissue engineering scaffolds can not only repair defective tissues from accidents/injuries, but also has the ability to treat disease untreatable with medicines [2]. The only thing that blocks the usage of scaffolds to regenerate large organs is the fact that vascularization is not sufficient in the construct. With the introduction of macro-/micro-channels within the scaffolds, this problem could be significantly improved. 3D channel network significantly contributes to the field of regenerative medicine, in particular to the regeneration of large, vascularized tissues. Channels have the potential to facilitate the perfusion of blood flow and recruitment of macrophages to effectively restore tissue formation and regenerate new tissue. This shows the importance of channels in tissue regeneration and the possibilities that they may bring in the future.

### 3.3. Limitations

Although the field of tissue engineering and scaffolds is extremely large and diverse, not much research has been done on the effects of macro-/micro-channels on vascularization and tissue regeneration as a whole. As a result, only 14 related studies were found that truly coincided with the topic of this literature review. In addition to this, no study had the exactly same topic, so comparisons between these studies may not be conclusive. For example, one study may have used the 3D printing method to create their scaffold, a 500 µm channel diameter, and experimentation on rats and another study may have used the sacrificial template method, a 250 µm channel diameter, and only cell culturing experimentation. It would be impossible to truly compare the results of these two experiments because they each have different methods for synthesizing the scaffold, have different scaffold materials and dimensions, and have different types of experimentation. As a result, no statistical analysis could be done comparing the results from different studies and connections between two different studies may be inconclusive.

### 3.4. Future Studies

While reading this literature review, it is obvious that the incorporation of channels to scaffolds is still a relatively new idea. Currently, most of the studies are still either in the stage of experimentation on small animals or in the stage of in vitro cell culturing experimentation. As a result, most of the studies still attempted to prove the concept of the macro-/microchannel function through either in vitro cell culture or in vivo experimented on small organisms such as rats or rabbits. With the development of the new technologies for the preparation of channels in scaffolds constantly evolving, new studies need to be performed on the function of different channel configurations, channel densities in the porous scaffold, and channel diameters on the vasculature formation and immune regulation in vivo. The mechanistic studies on how channels stimulate vasculature formation and recruit/polarize immune cells would be needed to better understand the function of macro-/micro-channels in a porous scaffold. These functions would further regulate the tissue regeneration ability of the scaffold. The incorporation of macro-/micro-channels would brighten the future of tissue engineering. In the future, to make the application of channels more clinically relevant, more in vivo experiments need to be further performed on large animal models similar to humans, and eventually on humans.

## 4. Conclusions

The incorporation of macro-/micro-channels is beneficial to cell and growth factor recruitment, enhances vascularization, and reduces immune response regardless of tissue types and research model. The ideal size of channels may vary depending on the tissue type. It can be concluded that a macro- or large micro-channel is desirable for bone regeneration and a small micro-channel favors myocardial tissue engineering while millimeter size is needed to achieve blood perfusion function. Also, 3D printing and sacrificial template are the most frequently used methods in fabricating channeled scaffolds. All in all, the incorporation of channels into scaffolds is a new and exciting area or research that may translate into a treatment solution for the regeneration of large tissue defects.

## Figures and Tables

**Table 1:** List and overview of articles selected for review.

Scaffold Material	Channel Type	Fabrication Method	Target Application and Research Model	Reference
Polylactic acid (PLA) and nanocrystalline hydroxyapatite (nHA)	500 μm and 250 μm diameter microchannels	3D printing	Bone tissue engineering; In vitro cell culture: human mesenchymal stem cell (hMSC) and human umbilical vein endothelial cell (HUVEC)	[34]
Calcium phosphate cement (CPC)	Interconnected hollow channels of 500 μm and 250 μm sizes	Dissolution of gelatin fibers with diameters of 255 μm and 507 μm	Bone tissue engineering; In vivo rat subcutaneous implantation	[35]
Polycaprolactone (PCL)	Large pores with microchannels (μCh) 12.9 μm in bulk 21.1 μm on surface	3D printing with camphene and then camphene sublimed	Bone tissue engineering; In vitro cell culture: hMSC and HUVEC; In vivo rat subcutaneous implantation; In vivo rat calvarium defect model	[36]
Agarose hydrogel	Unidirectional longitudinal channels with diameters of 500 μm and a centre-centre spacing of 1 mm	Fabricated with a pillared polydimethylsiloxane array structure	Bone tissue engineering; In vivo mouse subcutaneous transplantation	[37]
Silicate bioceramic	Hollow struts with an external diameter of 1 mm and internal diameter of 500 μm	Coaxial 3D printing with a modified core/shell printer nozzle	Bone tissue engineering; In vitro cell culture: Rabbit BMSCs and HUVEC In vivo rabbit radius segmental defect model	[38]
Porous β-TCP	1 mm diameter hollow channels	β-TCP slurry casted in paraffin-beads filled mold, solidified, dried and sintered	Bone tissue engineering; In vitro cell culture: hBMSC In vivo canine mandible bony defect model	[39]
Chitosan and collagen	The main channel and the branch channel of approximately 744 and 580 μm in diameter	3D printed sacrificial carbohydrate template placed in the mold and dissolved after scaffold preparation	Myocardial tissue regeneration; In vitro cell culture: Rat cardiomyocytes, HUVEC and C2C12	[40]
Macroporous alginate scaffolds	An array of parallel channels with 200 μm diameter and 400 μm wall-to-wall spacing with shifting between lines of 300 μm	Laser piercing technique	Myocardial tissue regeneration; In vitro cell culture (rat cardiac cells and HUVEC) and in vivo mouse subcutaneous implantation	[41]
Gelatin hydrogel	2 mm interconnected channels	3D printed poly(vinyl alcohol) (PVA) sacrificial template	Prevasculature and blood perfusion; In vitro cell culture (HUVEC)	[42]
Gelatin hydrogel	Micro- and macro-channels of 16.37 and 150.46 μm in diam-eter	Poly(N-isopropylacrylamide) (PNIPAM) temperature-dependent water-soluble fibers	Prevasculature and blood perfusion; In vivo mouse and porcine models of hindlimb ischemia and in vivo mouse non-ischemia model (greater omentum, normal hindlimb, subcutaneous site)	[43]
Silk scaffold	254 μm or 508 μm hollow channels	Linear wire arrays (LWAs) of either 254 μm or 508 μm diameter arranged into a grid pattern with 1 mm spacing between the wires	Neo-vascularization and large tissue construct engineering; In vitro cell culture (human dermal neonatal fibroblasts) and in vivo mouse subcutaneous im-plantation	[10]
Chitosan scaffold	Regularly-oriented micro-channels (*φ* 500 μm), which differed for the inter-channel spacing, at 600, 700, or 900 μm	Aluminum grids with predetermined holes	Neo-vascularization and large tissue construct engineering; In vitro cell culture (mouse endothelial cells) and in vivo mouse subcutaneous implantation	[44]
Extracellular matrix (ECM-C)	Uniformly distributed parallel microchannels with an average diameter of 146 μm based on the longitudinal cross-section.	Implanted Aligned PCL microfibers in rat to generate fibrous tissue, then removed PCL and cellular component	Multi tissue regeneration, in vitro cell culture (rat skeletal muscle cells, Schwann cells, vascular smooth muscle cells), in vivo multi-site implantation (Skeletal muscle, nerve and vascular regeneration).	[45]
Poly (L-lactide-co-ε-caprolactone 50:50) (PLCL) nanofiber and polydioxanone (PDS) microfiber	240 μm channel diameter, inter-fiber pore size of 4.1 μm	Combining melt spinning and electrospinning methods	Nerve regeneration, in vitro cell culture (murine macrophage cells and Schwann cells), and in vivo rat sciatic nerve defects model.	[46]

## Data Availability

The data presented in this study are from publications with copyright permission and are available on request from the corresponding author.
